# Genetic Interactions Underlying the Biosynthesis and Inhibition of β-Diketones in Wheat and Their Impact on Glaucousness and Cuticle Permeability

**DOI:** 10.1371/journal.pone.0054129

**Published:** 2013-01-17

**Authors:** Zhengzhi Zhang, Wei Wang, Wanlong Li

**Affiliations:** 1 Department of Biology and Microbiology, South Dakota State University, Brookings, South Dakota, United States of America; 2 Department of Plant Science, South Dakota State University, Brookings, South Dakota, United States of America; Nanjing Agricultural University, China

## Abstract

Cuticular wax composition greatly impacts plant responses to dehydration. Two parallel pathways exist in Triticeae for manipulating wax composition: the acyl elongation, reduction, and decarbonylation pathway that is active at the vegetative stage and yields primary alcohols and alkanes, and the β-diketone pathway that predominates at the reproductive stage and synthesizes β-diketones. Variation in glaucousness during the reproductive stage of wheat is mainly controlled by the wax production genes, *W1* and *W2*, and wax inhibitor genes, *Iw1* and *Iw2*. Little is known about the metabolic and physiological effects of the genetic interactions among these genes and their roles in shifting wax composition during plant development. We characterized the effect of *W1, W2, Iw1,* and *Iw2* and analyzed their interaction using a set of six near-isogenic lines (NILs) by metabolic, molecular and physiological approaches. Loss of functional alleles of both *W* genes or the presence of either *Iw* gene depletes β-diketones and results in the nonglaucous phenotype. Elimination of β-diketones is compensated for by an increase in aldehydes and primary alcohols in the *Iw* NILs. Accordingly, transcription of *CER4-6*, which encodes an alcohol-forming fatty acyl-CoA reductase, was elevated 120-fold in *iw1Iw2*. *CER4-6* was transcribed at much higher levels in seedlings than in adult plants, and showed little difference between the glaucous and nonglaucous NILs, suggesting that *Iw2* counteracts the developmental repression of *CER4-6* at the reproductive stage. While *W1* and *W2* redundantly function in β-diketone biosynthesis, a combination of both functional alleles led to the β-diketone hydroxylation. Consistent with this, transcription of *MAH1-9*, which encodes a mid-chain alkane hydroxylase, increased seven-fold only in *W1W2*. In parallel with the hydroxyl-β-diketone production patterns, glaucousness was intensified and cuticle permeability was reduced significantly in *W1W2* compared to the other NILs. This suggests that both *W1* and *W2* are required for enhancing drought tolerance.

## Introduction

The aerial organs of terrestrial plants are coated with an extracellular layer of hydrophobic lipids, termed cuticle. Produced by epidermal cells, the cuticle plays important roles in plant growth and development and, as the interface between sessile plants and the environments they live in, in the interaction with abiotic and biotic elements [1], [2]. Based on solubility in organic solvents, the cuticle is composed of insoluble cutin and soluble cuticular wax. Cutin, a cell wall-bound ester polymer of modified fatty acids and glycerol, serves as the backbone of the cuticle [3], [4], [5]. Intracuticular wax is embedded in the underlying cutin framework and epicuticular wax is overlaid on the cutin matrix and intracuticular wax [2], [6]. Wax composition varies with developmental stage, between organs, and with genetic and environmental conditions [1], [4], [6], causing the plant to be bluish-white (glaucous) or nonglaucous. Glaucousness is the visible form of densely distributed epicuticular wax crystallites.

Our current knowledge of wax biosynthesis (Figure S1), which is mainly derived from the model plant Arabidopsis, indicates that this process begins with the release of C_16_ and C_18_ fatty acids from the acyl carrier protein (ACP) by fatty acyl–ACP thioesterase B (FATB) [7] and their subsequent activation to CoA thioesters by a long-chain acyl-CoA synthetase (LACS) [8]. The activated forms of these fatty acids are transferred from plastids to the endoplasmic reticulum (ER), where they are made available for fatty acid elongase (FAE), which extends them to very-long-chain fatty acids (VLCFAs). The FAE complex consists of four types of enzymes: β-keto acyl-CoA synthases (KCS), β-keto acyl-CoA reductase (KCR), 3-hydroxy-acyl-CoA dehydratase (HCD), and enoyl-CoA reductase (ECR). In Arabidopsis, *ECERIFERUM 6* (*CER6*) [9], [10], [11] and *KCS1* [12] encode KCS and *KCR1* [13], *PAS2* [14], and *CER10* [15] encode KCR, HCD, and ECR, respectively.

The resulting VLCFAs can be released from the FAE complex as free fatty acids, or converted either to primary alcohols by acyl reduction [16] or to alkanes by acyl decarbonylation through an aldehyde intermediate [6], [17] and further to secondary alcohols and ketones by hydroxylation [18]. In the acyl reduction branch, fatty acyl-CoA reductase CER4 exclusively reduces VLCFAs to the corresponding primary alcohols [19], and wax ester synthase/acyl-CoA:diacylglycerol acyltransferase 1 (WSD1) uses the long-chain and very-long-chain primary alcohols and C_16_ fatty acid for wax ester production [20]. In the decarbonylation branch, CER1 physically interacts with the wax-associated protein CER3 and ER-localized cytochrome b5 isomers to catalyze the redox-dependent biosynthesis of alkanes [21]. Subsequently, midchain alkane hydroxylase 1 (MAH1), a cytochrome P450 enzyme, oxidizes the alkanes to generate secondary alcohols and ketones [18].

All of the cuticular lipid species synthesized in the ER need to be deposited extracellularly. The ABC transporters ABCG12/CER5 [22] and ABCG11/WBC11 [23], [24] are involved in exporting wax through the plasma membrane to the apoplastic space. A glycosylphosphatidylinositol-anchored lipid transfer protein, LTPG, functions as part of the machinery, either as a regulatory component or by creating the appropriate conditions for cuticular lipid exportation [25]. Recent efforts have started to shed light on the regulatory network underlying wax production by identifying transcription factors involved in this process. Several transcription factors of the AP2/EREBP [26], [27], [28], [29] and MYB family [30], [31], [32], [33], [34] regulate genes involved in cuticle biosynthesis. CER7, a 3′-to-5′ exoribonuclease, conditions wax synthesis by degrading a specific mRNA species of a *CER3* repressor [35], [36].

In grasses, transposon tagging identified several maize *GLOSSY* (*GL*) loci. *GL1* is homologous to *CER3*/*WAX2* [37], [38], *GL2* is homologous to *CER2* [39], *GL4* is homologous to *CER6/CUT1* [40], and *GL8* is homologous to *KCR* [41], [42], [43]. More recently, screening of a rice T-DNA tagging population isolated a *CER6*/*CUT1* homolog, *WSL1* [44]. Furthermore, *GL15,* which encodes a transcription factor of the AP2 family and is involved in the transition from juvenile to adult leaves [45], and *OCL1,* which encodes a HD-ZIP IV family transcription factor that activates a fatty acyl-CoA reductase and a putative wax transporter [46], [47], were found to regulate cuticular wax biosynthesis.

In the tribe Triticeae, including wheat and barley, the acyl elongation, reduction, and decarbonylation pathway for the synthesis of alcohol-rich waxes is active in the vegetative stages. A parallel pathway, which predominates in the reproductive stages, is responsible for biosynthesis of β-diketones, i.e., hentriacontane-14,16-dione and its hydroxyl derivatives [48], [49], [50]. The β-diketone pathway differs from the acyl elongation, reduction, and decarbonylation pathway in substrate and inhibitor specificity [49] and in pathway-specific genes [51]. Because β-diketones are not detected in wax extracts from the model plants Arabidopsis and rice, the β-diketone biosynthetic pathway draws much less attention.

Common wheat or bread wheat (*Triticum aestivum* L) is an important, widely adapted crop that is cultivated in many arid or semiarid areas. The inheritance of wheat glaucousness is mainly governed by two sets of dominant genes, *W1* and *W2,* which promote glaucous formation, and *Iw1* and *Iw2,* which inhibit it. *W1* and *Iw1* are located on the short arm of chromosome 2B (2BS), and *W2* and *Iw2* on 2DS [52]. Genetic mapping using molecular markers has localized the *Iw1* [53] and *Iw2* locus [54], [55] to the distal regions of the chromosome arms. While the non-glaucous phenotype prevails in the wild diploid ancestors [52], [56], the glaucous type predominates in cultivated polyploid wheat, suggesting that glaucousness was favored during wheat domestication. Field physiology studies showed that glaucousness is positively related to wheat yield, especially under drought-stressed conditions [57], [58], [59]. Despite its adaptive importance, the molecular mechanisms underlying variation in glaucousness and its association with drought tolerance remain largely unknown. We characterized a set of six near isogenic lines (NILs) by scanning electron microscopy (SEM), metabolite profiling of wax extracts, measuring the rate of water loss and chlorophyll efflux, and transcriptional profiling of 72 cuticle genes. Here, we report the results of this study and their implications in our understanding of cuticular wax pathways and drought tolerance.

## Results

### Genetic Background Analysis of Wax NILs

**Figure 1:**
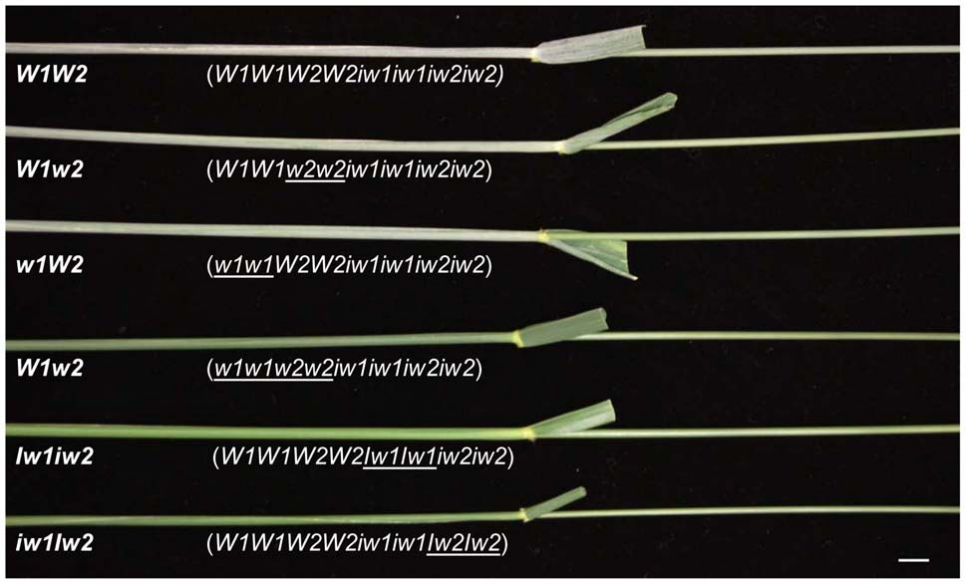
Flag leaf sheaths and peduncles of the NILs examined in this study. NIL designations are indicated beneath each peduncle and genotypes are specified in parentheses. The introduced alleles in the genotypes are underlined. The bar indicates 1 cm.

Six wax NILs were developed by ten backcrosses to wheat cultivar S-615 [52] and their genotypes and glaucousness on flag leaf sheaths and peduncles are shown in Figure 1. The NILs *W1W2*, *W1w2,* and *w1W2* are glaucous, whereas *w1w2*, *Iw1iw2* and *iw1Iw2* are not, indicating that *W1* and *W2* are functionally redundant in forming glaucousness, but that a single *Iw* gene is sufficient to suppress glaucousness formation. Before physiological, metabolic, and expressional characterization, we inspected the genetic background by single nucleotide polymorphism (SNP) and simple sequence repeat (SSR) genotyping. Of the 9,000 SNP probes assayed, over 5,200 produced positive signals in each line and 18 probes (0.35% of 5,200) or fewer detected polymorphisms among the NILs, suggesting that the NILs are identical over 99.5% of the genome. The lowest level of polymorphism was found between NILs *W1W2* and *iw1Iw2*, where only one probe detected a polymorphism. We also genotyped the six NILs using SSR markers previously mapped at the tips of 2BS and 2DS arms, where the *W* and *Iw* genes are located. All of the NILs were positive for all SSR markers, indicating that the mutations were not caused by chromosome deletions. These results indicate that the NILs differ from each other only at a very small fraction of the genome and suggest that there are probably no polymorphisms in wax genes other than at the *W* and *Iw* loci.

### Wax Morphology

**Figure 2:**
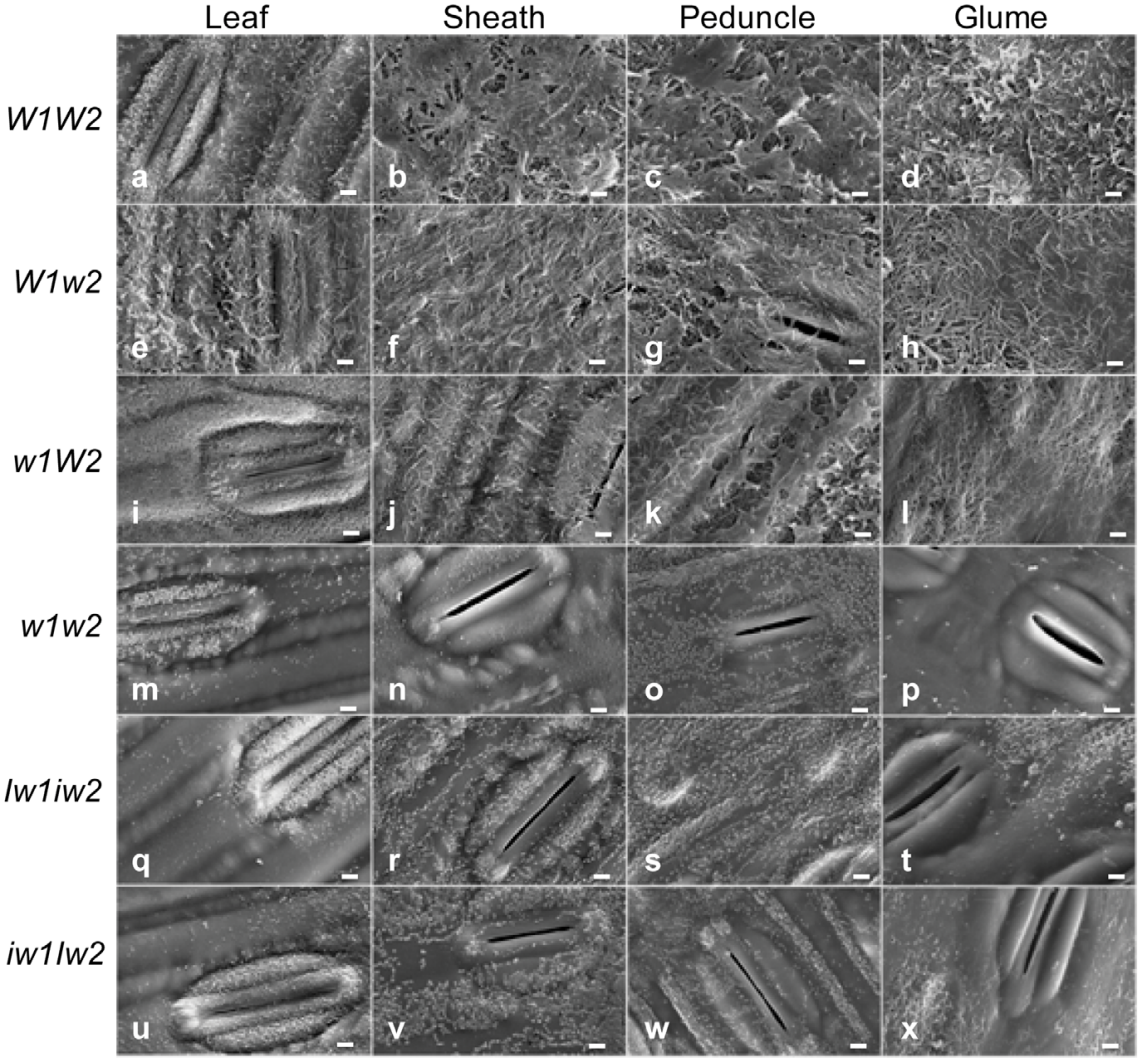
Electron micrographs of the cuticle surfaces of flag leaf blades, sheaths, peduncles, and glumes of the NILS. The tissues are indicated on the top and the NIL designations at the left. The bars indicate 2.5 µm.

When flowering begins at Fakes’ stage 10.5.1 (F10.5.1), glaucousness is fully developed in the upper part of the wheat plant. To gain insight into the organization of epicuticular wax, we examined the wax crystallites deposited on the abaxial surface of the flag leaf blades and sheaths, the peduncles, and the glumes of the NILs at this stage under SEM. The morphology of wax crystallites in glaucous NILs was clearly different from that of nonglaucous ones (Figure 2). In all tissue types, the cuticle surfaces of glaucous NILs were covered with a meshwork of wax tubes, and the density was higher in *W1W2* (Figure 2a, 2b, 2c, and 2d) and *W1w2* (Figure 2e, 2f, 2g, and 2h) than in *w1W2* (Figure 2i, 2j, 2k, and 2l). Besides wax tubes, large wax sheets were also observed above the wax meshwork in the sheath and peduncle of the *W1W2* plants (Figure 2b and 2c). On the leaf cuticles of the *w1W2* plants, sparse wax tubes were seen over the ground cells, whereas fine wax granules densely covered the guard cells (Figure 2i).

In contrast to the glaucous NILs, the cuticles of the nonglaucous NILs were either smooth (Figure 2p) or carried fine wax particles. On the leaf cuticles, wax crystallites were mainly found over the guard cells (Figure 2m, 2q, and 2u). However, the guard cells of glumes of the *Iw* NILs were devoid of wax crystallites (Figure 2t and 2x). Wax crystallites were distributed on both ground and guard cells in the sheath (Figure 2n, 2r and 2v) and peduncle cuticles (Figure 2o, 2s and 2w).

Wax morphology is determined by wax composition. The dramatic difference in wax morphology suggests possible differences in wax content, composition, and cuticle permeability between the glaucous and nonglaucous NILs. Considering that sheath wax morphology was a good indicator of the differences among the NILs, we concentrated our research on the sheath wax.

### Wax Composition

**Figure 3:**
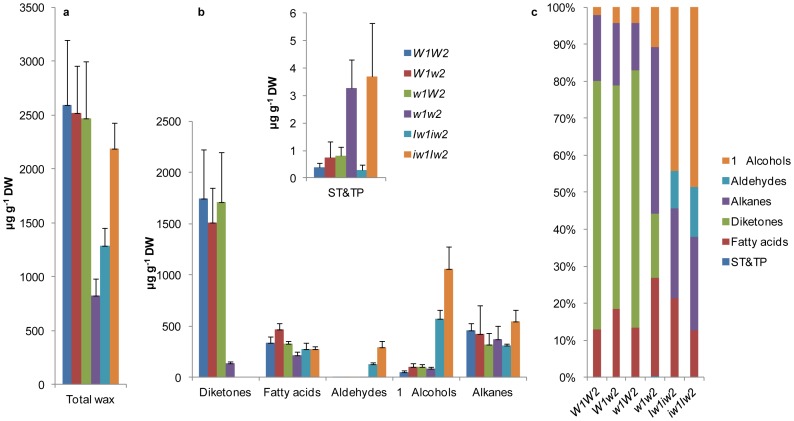
Wax composition of the six NILs. (a) Total wax load of the flag leaf sheaths was measured by GC-MS. (b) β-diketone, fatty acid, aldehyde, primary (1°) alcohol, alkane, and sterol and triterpene (ST&TP) content. The numbers on the y-axes indicate average content expressed as µg per g dried tissue (dry weight, DW). The bars indicate standard deviation of the mean estimated from six biological replicates. (c) The percentage of wax species in each genotype was calculated from the means.

We extracted cuticular wax from the flag leaf sheaths of the wax NILs at stage F10.5.1 and profiled it using gas chromatography-mass spectrometry (GC-MS). The results showed that ∼90% of the wax extract consisted of known wax species and the remaining ∼10% consisted of unknown species. As expected, the NILs varied greatly in total wax load and composition. All glaucous NILs carried the same wax load *(P*>0.82980; Figure 3a) and had a similar wax composition, with β-diketones accounting for >60%, fatty acids and alkanes for ∼15% each, primary alcohols for 2 to 4%, and cyclic lipids for trace amounts of total wax (Figures 3b and 3c). Wax load varied by ∼60% among the nonglaucous NILs. Compared to *W1W2*, no significant reduction was found in *iw1Iw2* (*P = *0.42065); however, a 50% and 68% reduction was detected in *Iw1iw2* (*P = *0.0412) and *w1w2* (*P = *0.01577), respectively (Figure 3a). This indicates that wax composition, instead of wax load, determines glaucousness. In contrast to the glaucous NILs, β-diketones were depleted in the nonglaucous NILs, being reduced to ∼8% of total wax species in *w1w2* (*P = *0.00925) and completely eliminated in *Iw1iw2* and *iw1Iw2* (Figure 3c). There was little net increase in *w1w2* for the remaining components (*P*>0.12834), which elevated the alkane proportion to ∼45% of the total wax species (Figure 3c), indicating that *W* genes are specific for β-diketone synthesis. In contrast to the situation in *w1w2*, loss of β-diketones in *Iw1iw2* and *iw1Iw2* was compensated by an increase of aldehydes and primary alcohols. Compared to *W1W2*, aldehydes and primary alcohols were increased ∼250-fold and ∼10-fold in *Iw1iw2*, and ∼600-fold and 20-fold in *iw1Iw2*, respectively (*P*<0.00254; Figure 3b). This indicates that the *Iw* genes inhibit the synthesis of β-diketones and shunt their substrates to the fatty acyl reduction pathway. In this respect, the action of *Iw2* is much stronger than that of *Iw1*. As a result, three different types of waxes are recognized in this NIL set: β-diketone-rich wax in the glaucous NILs, alkane-rich wax in *w1w2*, and primary alcohol-rich wax in *Iw1iw2* and *iw1Iw2* (Figure 3c). In contrast to the aliphatic wax species, cyclic wax species, such as sterols and triterpenes (ST&TP) were detected at much lower levels. Compared to *W1W2*, ST&TP, which mainly consisted of β-sitosterol and β-amyrin, were increased nine-fold in *w1w2* (*P = *0.0171; Figure 3b).

**Figure 4:**
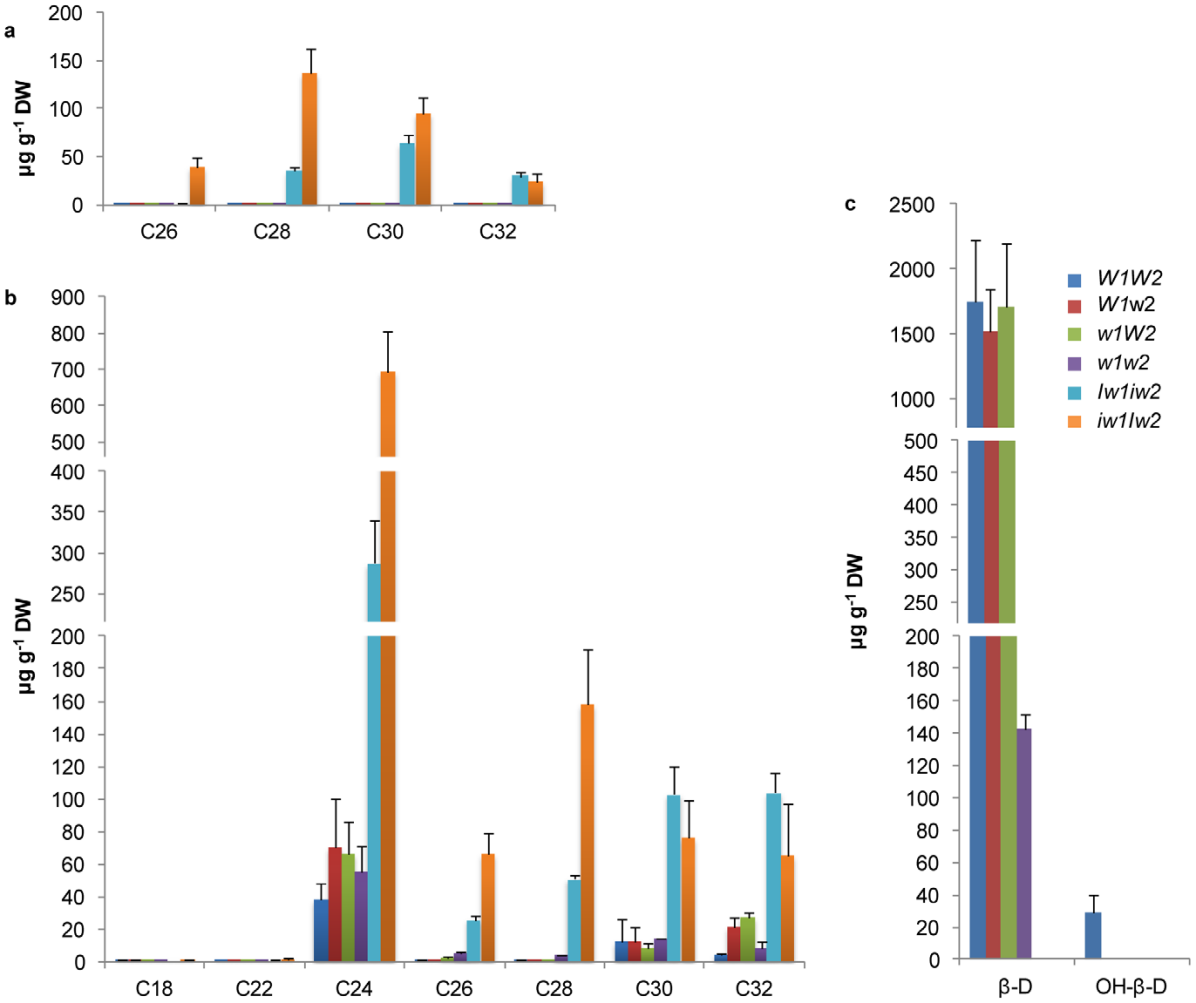
Homolog variation of major wax species. Carbon atom numbers of aldehydes (a) and primary (1°) alcohols (b), and β-diketones (c) are indicated on the x-axes. Their contents are indicated on y-axes as µg per g dried tissue. The bars indicate standard deviation of the mean calculated from six biological replicates. β-D, β-diketone; and OH-β, hydroxyl-β-diketones.

The carbon chain length ranges from C_12_ to C_32_ in fatty acids, C_25_ to C_31_ in alkanes (Figure S2), C_26_ to C_32_ in aldehydes, and C_18_ to C_32_ in primary alcohols (Figure 4a and 4b). Compared to *W1W2*, aldehyde homologs were increased by ∼14- to 485-fold in *Iw1iw2* and by ∼170- to 1110-fold in *iw1Iw2* (*P*<0.00928; Figure 4a). VLCFA-derived primary alcohol homologs were increased by 7- to 75-fold in *Iw1iw2* and by 6- to 112-fold in *iw1Iw2* (*P*<0.03231; Figure 4b). Although tetracosan-1-ol (C_24_) was the most abundant homolog, the maximal increase was found in octocosan-1-ol (C_28_) in *Iw1iw2* and in hexacosan-1-ol (C_26_) in *iw1Iw2* (Figure 4b).

A single carbon chain length, C_31_, was detected for β-diketones; however, three substituted derivatives were identified: 8- and 9-hydroxyl isomers, and enolic isomer. Considering keto-enol tautomerism, we combined enolic β-diketone with β-diketone. No difference in the unsubstituted β-diketone was found among the glaucous NILs; however, that amount was reduced >10-fold in *w1w2* compared with in the glaucous NILs (*P*<0.00985; Figure 4c). Hydroxyl-β-diketones were only detected in *W1W2*, and not in *W1w2* and *w1W2* (Figure 4c), indicating that an interaction between *W1* and *W2* is required for β-diketone hydroxylation at C_8_ or C_9_. Depending on the presence of hydroxyl β-diketones, the β-diketone-rich wax can be further divided into two types, OH-β wax in *W1W2* and β-D wax in *W1w2* and *w1W2*.

### Transcriptional Profiling of Cuticle Genes

When flag leaves are fully emerged from the whorl (stage F9.0), flag leaf sheaths elongate rapidly. Because cuticle genes are highly expressed in the elongating epidermal cells [60], [61], we selected flag leaf sheaths at this stage for transcription profiling. We chose nine candidate reference genes (Table S1) from previous publications [62], [63], [64] and validated their expression stability in 18 cDNA samples from the NIL set using programs qBase^Plus^ (Biogazelle, Belgium) and NormFinder [65]. Both programs demonstrated that *TaRPII36* was the best reference gene for quantifying cuticle gene expression (Figure S3).

**Figure 5:**
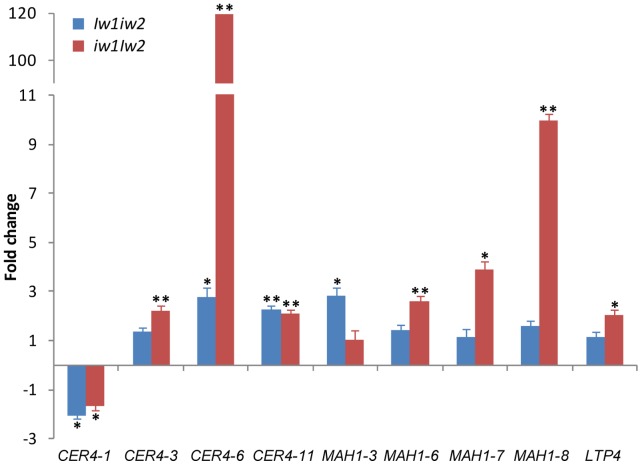
Expression of cuticular wax genes in the wheat flag leaf sheath of the *Iw* NILs compared to *W1W2*. Genes with two- or higher-fold changes are depicted and expression data for all genes analyzed are listed in Table S2. The bars represent standard deviation of the mean fold-change of mRNA levels. Asterisks indicate that the difference is significant at *P*<0.05 (*) or at *P*<0.01 (**).

The increase of aldehydes and primary alcohols in *Iw1iw2* and *iw1Iw2* suggests that the *Iw* genes activate *CER4* homologs; the abundance of β-diketones in the glaucous NILs suggests that the *W* genes up-regulate special *KCS* homologs and the decarbonylation components and the *Iw* genes suppress them; and the presence of hydroxyl-β-diketones only in *W1W2* suggests that the interaction between *W1* and *W2* activates *MAH1* homologs. To test these hypotheses, we profiled the transcription of wheat wax candidate genes with an emphasis on the *CER1*, *CER3*, *CER4*, *KCS,* and *MAH1* gene families. To test if cutin is also involved in the phenotype variation, we selected five cutin biosynthetic genes. We designed primers for 64 unigenes and adopted primers for eight additional genes from a previous publication [66]. Thus, we evaluated the transcription of 72 genes involved in cutin and wax biosynthesis, transport, and transcription regulation (Table S1). Compared with *W1W2*, the expression of 11 genes in *Iw1iw2* and 29 genes in *iw1Iw2* was significantly altered, of which the expression patterns of seven genes were shared by *Iw1iw2* and *iw1Iw2* (Table S2). Of these 72 genes examined, nine, including four *CER4* members, four *MAH1* members, and *LTP4,* showed over a two-fold difference in expression. The most dramatic change was observed in *CER4-6*, which was up-regulated ∼120-fold in *iw1Iw2* and 2.8-fold in *Iw1iw2* (Figure 5). Searching the D-genome physical mapping database (http://probes.pw.usda.gov/WheatDMarker) revealed that *CER4-6* is located on chromosome 5D. This indicates that *CER4-6* is not *Iw2* itself, which is located on chromosome 2D, but an *Iw2* target that possibly plays a role in increasing aldehyde and primary alcohol content. *CER4-11* was also up-regulated two-fold in both *Iw1iw2* and *iw1Iw2*. In addition, four *MAH1* members, *MAH1-4*, *MAH1-7*, *MAH1-8,* and *MAH1-9*, were differentially expressed in *Iw1iw2* and *iw1Iw2* (Figure 5 and Table S2). Together, these results indicate that *Iw1* differs from *Iw2* in terms of the molecular mechanisms for increasing aldehyde and primary alcohol content.

**Figure 6:**
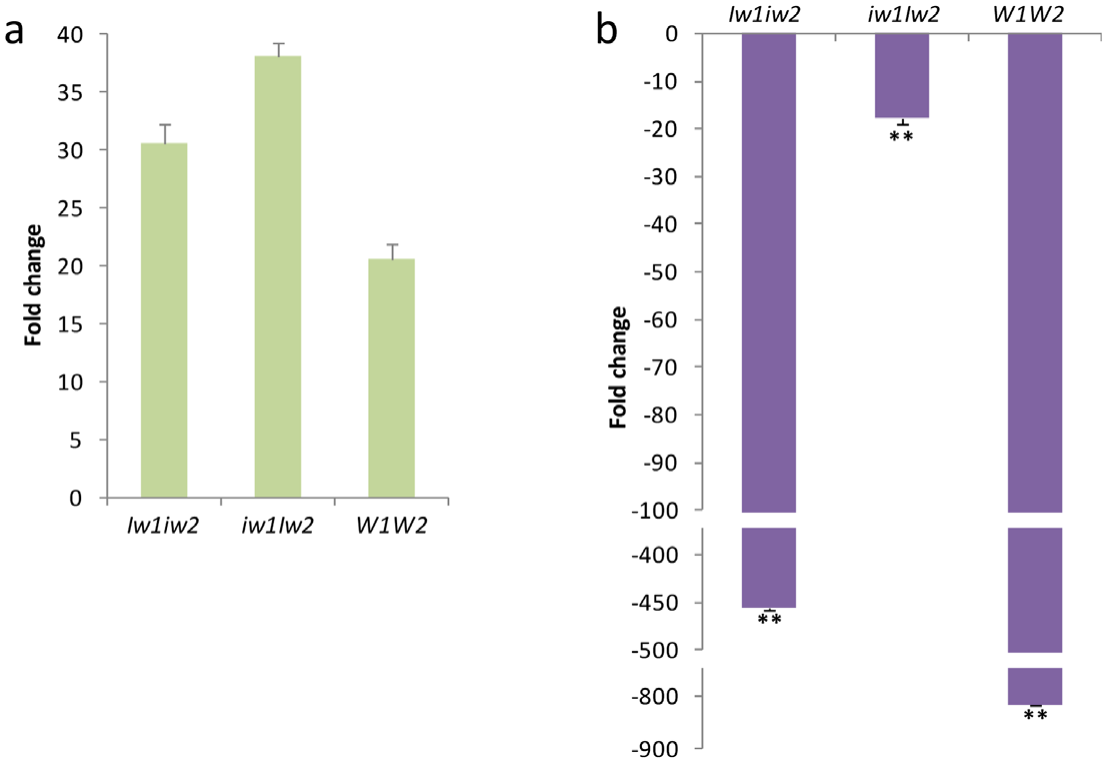
*CER4-6* expression in sheaths of *Iw1iw2*, *iw1Iw2,* and *W1W2* at different developmental stages. (a) Transcription levels of *CER4-6* at stage F4.0 compared to that of the reference gene *TaRPII36*. (b) Fold changes of *CER4-6* transcription at stage F9.0 compared to that at stage F4.0. The bars represent standard deviation of the mean fold-change of mRNA levels. Asterisks indicate that the difference is significant at *P*<0.01 (**).

We further asked if the up-regulation of *CER4-6* was the result of activation or de-repression by *Iw2*. To test this, we examined the transcription of *CER4-6* in sheaths of *W1W2*, *Iw1iw2,* and *iw1Iw2* plants at the seedling stage (F4.0). Wax composition changed dramatically during wheat development, from alcohol-rich wax in the vegetative stage to β-diketone-rich wax in the reproductive stage, particularly in leaf sheaths [48]. We found that *CER4-6* transcription was increased by less than two-fold in *Iw1iw2* and *iw1Iw2* compared to *W1W2* at stage F4.0 (*P*<0.0002; Figure 6a), which was much smaller than the difference detected at stage F9.0 (Figure 5). Compared to stage F4.0, *CER4-6* was down-regulated 815-fold in *W1W2* (*P = *1×10^−7^) and 450-fold in *Iw1iw2* (*P = *2×10^−7^), but only 17-fold in *iw1Iw2* at stage F9.0 (*P = *4×10^−7^; Figure 6b). This indicates that expression of *CER4-6* is under developmental regulation and is suppressed at the reproductive stages, and that *Iw2* counteracts this developmental suppression.

**Figure 7:**
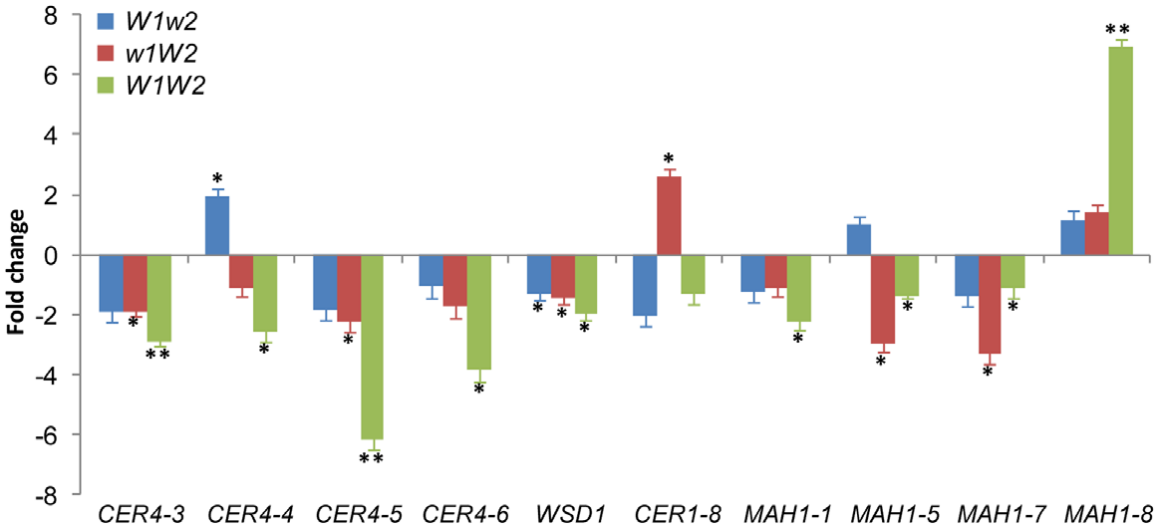
The expression of cuticular wax- and cutin-related genes in the wheat flag leaf sheath of *W1*w2, *w1W2,* and *W1W2* plants compared to *w1w2*. Genes with two- or higher-fold changes are depicted and expression data for all genes analyzed are listed in Table S3. The bars represent standard deviation of the mean fold-change of mRNA levels. Asterisks indicate that the difference is significant at *P*<0.05 (*) or at *P*<0.01 (**).

Compared to the situation in *w1w2*, expression of four genes in *W1w2*, 19 genes in *w1W2,* and 26 genes in *W1W2* was significantly altered (Table S3). The expression patterns of three genes overlapped between *W1w2* and *W1W2*, 15 between *w1W2* and *W1W2,* and two among all three glaucous NILs. While transcriptional changes of five genes relative to *w1w2* were detected in *W1w2* or *w1W2* but not in *W1W2*, changes of another 10 genes were detected in *W1W2* but not in *W1w2* or *w1W2*. This indicates that *W1* and *W2* had a non-additive effect on the expression of these cuticular wax-related genes. The expression of four genes in *w1W2* and six genes in *W1W2* was altered by two-fold or more, including four *CER4* members, five *MAH1* members, and *CER1-8* (Table S3; Figure 7). The expression of *MAH1-8* matches the production pattern of hydroxyl-β-diketones: no change in *W1w2* and *w1W2* were observed, but the expression increased seven-fold in *W1W2*. *MAH1* is responsible for alkane hydroxylation in Arabidopsis [18], suggesting a role for this gene in generating hydroxyl-β-diketones in wheat.

### Cuticle Permeability

Nonstomal transpiration is directly correlated with cuticle permeability. We evaluated the cuticle permeability of this NIL set by measuring the rates of water loss and chlorophyll efflux of the flag leaf sheaths at stage F10.5.1. Simultaneously, we inspected stomatal density and aperture size under a light microscope. All of the NILs had a similar stomal density, i.e., ∼60 stomata in a field of view at a magnification of 10×20, and most stomata were closed 1 h after detachment at room temperature and under lab conditions. Therefore, the differences observed in water-loss rate were presumably attributed to cuticle permeability.

**Figure 8:**
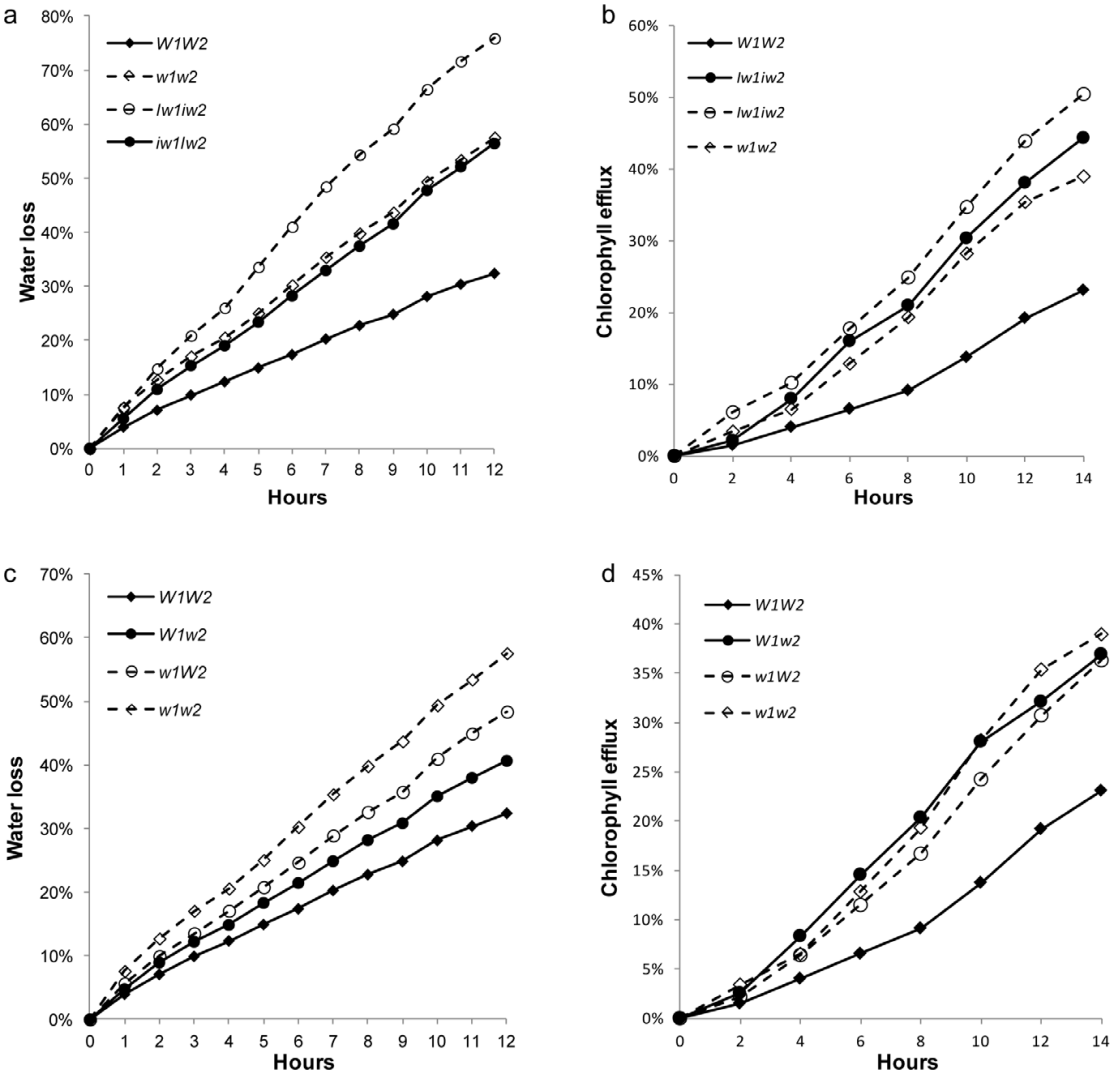
Cuticle permeability of the NILs. Cuticle permeability was evaluated by air drying at room temperature (a and c) and chlorophyll leaching in 80% ethanol (b and d). The numbers on the x-axes represent hours of treatment. Water loss or chlorophyll leaching at each time point is represented on the y-axes as percentages of the total water content or total chlorophyll content in the tissue. Measurements taken from four individuals were averaged.

Compared to *W1W2*, *Iw1iw2* and *iw1Iw2* started showing higher rates of water loss 1 h after detachment (*P*<0.03694) and the differences remained and even increased thereafter (Figure 8a). The *Iw* NILs also showed significantly higher chlorophyll leaching rates than *W1W2* after 4 h of treatment (*P*<0.04285; Figure 8b). Although *Iw1iw2* and *iw1Iw2* exhibited similar chlorophyll efflux rates (*P*>0.05503; Figure 8b), *Iw1iw2* showed a higher water-loss rate after 4 h of treatment (*P*<0.03272; Figure 8a). These findings echo the wax data and suggest that the increased amount of primary alcohols and aldehydes in *iw1Iw2* have some effect on blocking nonstomatal transpiration, but not on preventing chlorophyll efflux.

Similar to *iw1Iw2*, *w1w2* showed a significantly higher rate of both water loss and chlorophyll leaching compared to *W1W2* (Figure 8a and 8b). The difference in water loss between *w1w2* and *W1W2* was detectable as early as 1 h after detachment (*P = *0.03607; Figure 8c) and that in chlorophyll leaching at 6 h of treatment (*P = *0.04527; Figure 8d). In both experiments, the performance of *w1W2* was similar to that of *w1w2* (*P*>0.13101) and significantly different from *W1W2* after 3 h of desiccation and 6 h of chlorophyll-leaching (*P*<0.0494). The findings for *W1w2* were not consistent in the two experiments. The water-loss rate of this NIL was intermediate, being higher than that of *W1W2* (*P*<0.04174), but lower than that of *w1w2* (*P*<0.0356 (Figure 8c). In the chlorophyll-leaching experiment, *W1w2* showed high similarity to *w1w2* (*P*>0.11128) and significant difference from *W1W2* after 6 h of treatment (*P*<0.02202; Figure 8d). Collectively, *W1W2* showed significantly lower rates of water loss and chlorophyll efflux than the nonglaucous and other glaucous NILs, suggesting that cuticle permeability is not inversely proportional to wax load, but rather that it is closely related to wax composition, and that *W1* and *W2* function epistatically to reduce cuticular transpiration and chlorophyll leaching.

## Discussion

Glaucousness is a classic genetic marker trait of wheat. Recent drought threat renewed interest in this trait. Despite several reports on the molecular mapping of *Iw* genes [53], [54], [55] and quantitative trait loci that affect glaucousness [67], this is the first systematic characterization of wheat glaucousness. In this study, we combined SEM, metabolite profiling, gene expression profiling, and physiologic approaches in a near isogenic background to gain insight into the genetic regulation of cuticular wax biosynthesis and its perturbation during drought tolerance.

### Wax Composition and Glaucousness

Numerous cuticular wax genes were identified based on the loss- or reduction-of-glaucousness phenotypes, such as the *cer* mutants of Arabidopsis. The *CER* genes participate in almost all steps of cuticle biosynthesis, indicating that glaucousness formation requires multiple wax components. Comparative analyses of the cuticular wax profiles of glaucous and nonglaucous durum lines and of wheat aneuploids found that β-diketone was critical for glaucousness formation [68], [69]. Our research showed that wax composition is more important than wax load in glaucousness determination and that the nonglaucous NILs differ from the glaucous ones in terms of β-diketone content. *Iw1iw2* and *iw1Iw2* completely lacked β-diketones and *w1w2* exhibited a ∼90% reduction in β-diketones. These findings confirm that β-diketones are essential for glaucousness formation. SEM observation also confirmed that wax composition was directly related to wax morphology: β-diketones form wax tubes in the glaucous NILs and primary alcohols form wax chips in the *Iw* NILs. The glaucous NILs and *w1w2* carried trace aldehydes and low levels of primary alcohols, suggesting that these wax species play a limited role in glaucousness formation.

Compared to *W1w2* and *w1W2*, *W1W2* showed slightly higher levels of glaucousness (Figure 1), but no difference in total β-diketone load. The major difference among the glaucous NILs is the presence of 8- and 9-hydroxyl β-diketones in *W1W2* and the absence of these hydroxy isomers in *W1w2* and *w1W2*. It is believed that hydroxyl-β-diketone is derived from β-diketone through mid-chain hydroxylation. In barley, this hydroxylation is defined by the *cer-u* mutants [70]. In the genus *Triticum*, 25-isomer is the typical hydroxyl-β-diketone in durum wax, but is substituted by 8- and 9-isomers in hexaploid wheat [71], suggesting that the generation of these substituted β-diketones involves the interaction of several genes. The absence of hydroxyl β-diketones in *W1w2* and *w1W2* suggests that the *W1-W2* interaction is required for β-diketone hydroxylation and that these substituted β-diketones are important for intensifying glaucousness and forming wax crystallite sheets in *W1W2* (Figure 2b and 2c).

### Wax Pathways

During plant development, multiple wax pathways operate in parallel to produce different wax types [49], [51], [72], and wax composition changes as plants develop and grow. In the Triticeae, primary alcohols and alkanes are the predominant wax species at the seedling stage [61], [66] and β-diketones are abundant at the reproductive stage, particularly in the sheaths and spike [48], [61], [70]. Genetic and biochemical studies in barley supported the existence of two parallel pathways, the acyl elongation, reduction, and decarbonylation pathway and the β-diketone pathway [50]. Mutations in *CER-cqu* of barley [51] and in the *W* genes of wheat (Figure 3) specifically affect β-diketone synthesis. However, we know little about the cross-talk between these two pathways. In the present research, we identified three major types of waxes in a set of six NILs: alkane-rich wax in *w1w2*, alcohol-rich wax in the *Iw* NILs, and β-diketone-rich wax in the glaucous NILs (Figure 3c). A survey of Triticeae species found that many wild species produce alcohol-rich wax at the reproductive stage [73], implying that the *Iw* alleles prevail in nature and that the *iw* mutations led to the production of β-diketone-rich wax. The similar wax loads in *W1W2* and *iw1Iw2* (Figure 3a) suggest that precursors are shunted between the acyl reduction pathway and the β-diketone pathway by *Iw2*. Consistent with this, we found that expression of *CER4-6* was elevated 120-fold in *iw1Iw2*. The wax composition in the *Iw* NILs at the adult stage (F10.5.1) resembles that at the seedling stage [48], being rich in primary alcohols and lacking β-diketones. Transcription quantification further demonstrated that *CER4-6* is active in the seedling stage (F4.0) in *W1W2*, *Iw1iw2,* and *iw1Iw2* (Figure 6a). Expression of *CER4-6* was reduced in all three NILs at stage F9.0; however, the reduction was most pronounced in *W1W2* (Figure 6b). Similarly, expression of four FAE genes showed no difference between *iw1Iw2* and *W1W2* in the seedling stage, but was reduced at the adult plant stage to a much greater extent in *W1W2* than in *iw1Iw2* (Figure S4). This suggests that the genes involved in the acyl elongation and reduction pathway are repressed by reproductive development and de-repressed by the *Iw* genes, which act as cross-talkers that regulate the acyl reduction and β-diketone pathway. *Iw2* maintains an active *CER4-6* at the reproductive stage and suppresses β-diketone production. It would be fascinating to determine how these two opposite activities are coupled by one gene. This is probably achieved by an interaction between *Iw2* and different sets of genes. Molecular cloning of *Iw2* and an in-depth analysis of *CER4-6* expression will provide insight into the developmental regulation of the wax composition shift.

Much work has focused on the biosynthesis of β-diketones in barley by genetic and metabolic analyses of the *cer* mutants. Wax profiling found that *cer-q*, *cer-c,* and *cer-u* mutants define chain elongation, decarbonylation, and hydroxylation reactions in β-diketone synthesis, respectively [50], [70]. However, genetic analyses of these mutants in barley support the hypothesis that β-ketoacyl elongation, decarbonylation, and hydroxylation are carried out by one gene, *cer-cqu* [50], [51]. It is difficult to imagine how one gene might govern three different reactions. *cer-cqu* has been redesignated as *gsh1* and was mapped to the subtelomeric region of the barley chromosome arm 2HS (http://wheat.pw.usda.gov/ggpages/bgn/26/BGS351), which is colinear to the *W1* locus in wheat [52]. In the present research, we found that the *W1* and *W2* genes in wheat are each sufficient for the deposition of β-diketone and complement each other, but that both are required for the synthesis of hydroxyl-β-diketones. One scenario would be that the *W* genes encode transcription factors belonging to the same family of proteins that activate the transcription of the β-diketone biosynthetic genes. We profiled 18 *FAE* genes; however, none of them were upregulated in the glaucous NILs. This is probably because β-diketones are synthesized by a separate enzyme system [50], but that the genes tested were chosen based on their function in model plants, which do not synthesize β-diketones. The proposed β-diketone biosynthetic pathway includes a decarbonylation reaction [50]. We examined the expression of eight *CER1* and five *CER3* members of the decarbonylation pathway, and found that only the expression of *CER1-8* was increased in *w1W2* (2.6-fold). To explore the possibility that *MAH1* participates in β-diketone hydroxylation, we evaluated the expression of eight *MAH1* members and found that *MAH1-8* matched the production pattern of hydroxyl-β-diketones (Figures 4c and 7). Compared to *W1W2*, transcription of *MAH1-8* was further elevated (∼10-fold) in *iw1Iw2*, but hydroxyl-β-diketone was not detected, because *Iw2* inhibits the biosynthesis of β-diketone, the substrate of hydroxyl-β-diketones.

The wheat *W1* gene resembles the barley *cer-cqu* locus in terms of chromosomal location and regulation of β-diketone synthesis and hydroxylation. Considering the close phylogenetic relationship between barley and wheat, *cer-cqu* is probably orthologous to *W1* and may also need to interact with other genes for β-diketone hydroxylation.

### β-Diketones and Drought Tolerance

Previous physiological studies in wheat found that glaucousness significantly increased grain and biomass yield in irrigated and rainfed field experiments [74], and increased the photosynthesis to transpiration ratio and reduced the photosynthetic surface temperature in greenhouse experiments [58]. Glaucousness significantly reduced transpiration at night, which caused a relatively greater reduction in transpiration than photosynthesis and the increase of water-use efficiency [58]. In the present research, we measured cuticle permeability in terms of water loss and chlorophyll leaching in six NILs of four wax genes, and profiled their wax composition and inspected their wax morphologies. This allowed us to compare the effect of the individual genes and to analyze their interaction. We found that cuticle traits are closely associated with wax composition, mainly with respect to the β-diketones. The nonglaucous NILs had little or no β-diketones and showed significantly higher water-loss and chlorophyll-leaching rates (Figures 8a and 8b). A small but significant effect was also seen for other wax species. Although *Iw1iw2* and *iw1Iw2* had similar chlorophyll-leaching rates (fig. 8b), the former had a higher water-loss rate (Figure 8a), probably because *iw1Iw2* wax had a significantly higher content of primary alcohols, aldehydes, and alkanes (Figure 3). Cuticle trait differences were also observed among the glaucous NILs. *W1w2* and *w1W2* had higher chlorophyll-leaching and water-loss rates than *W1W2* (Figures 8c and 8d). The most significant difference in the glaucous NILs in terms of wax composition is the presence of 8- and 9-hydroxyl-β-diketones in *W1W2*, suggesting a role of these hydroxyl isomers in reducing cuticle permeability and improving drought tolerance. Compared to β-diketone, the hydroxyl-β-diketones were much less abundant in the sheath wax. It is hard to imagine how hydroxyl-β-diketones reduce cuticle permeability. One explanation is that the addition of hydroxyl-β-diketones may underlie the changes in wax crystal organization of *W1W2* (e.g., the formation of wax crystal sheets), which result in reduced water loss and chlorophyll efflux. We hypothesize that the hydroxyl-β-diketones function as the “glue” that cross-links the β-diketone tubes via the formation of hydrogen bonds between the hydroxyl and keto groups. Application of matrix-assisted laser desorption/ionization–MS imaging to the NILs and re-crystallization analysis of combinations of β-diketone and substituted β-diketone at different ratios may shed light on this possibility.

Glaucousness is an adaptive trait to dry cultivation conditions and will play an important role in developing cuticle-based strategies to improve drought tolerance. The *Iw* genes have a negative impact on drought tolerance and need to be eliminated from wheat breeding programs. Due to the dominant mode of inheritance, *Iw*-mediated nonglaucousness can be eradicated by one selection in early generations. The manipulation of the *W* genes is more challenging. Our results indicate that one *W* gene is sufficient to restore glaucousness, but not to prevent nonstomal transpiration. Therefore, both *W* genes are required for enhancing drought tolerance. In this respect, marker-assisted selection will help improve breeding efficiency. To this end, user-friendly molecular markers tightly linked to the *W* genes need to be developed. Molecular mapping and cloning of these *W* genes will open novel routes to manipulate cuticle permeability for drought tolerance.

### Conclusions

In summary, characterization of a set of six NILs demonstrated that β-diketones contribute to glaucousness formation in the reproductive stage. A single *W* gene is sufficient for β-diketone synthesis, but both *W1* and *W2* are required for β-diketone hydroxylation. The *Iw* genes suppress β-diketone synthesis, but promote the production of aldehydes and primary alcohols. Consistent with the wax profiles, *CER4-6* was de-repressed by *Iw2*, and *MAH1-8* was activated by the interaction between *W1* and *W2*. *W1W2* showed the lowest cuticle permeability, suggesting that hydroxyl-β-diketones play a role in drought tolerance.

## Materials and Methods

### Plant Materials and Growth Conditions

One set of six NILs in the S-615 background was developed by Tsunewaki and Ebana [52] and the seeds were obtained from the corresponding author. The NILs were planted in 4×4 inch pots containing Sunshine® Container Potting Mix 3 (Sun Gro Horticulture) supplemented with Multicote® 8 Controlled-Release Fertilizer (Haifa) and grown in a greenhouse at a temperature of 20°C in the day and 18°C at night and with a day length of 16 h. Total genomic DNA was isolated from the NIL set using the Plant DNeasy Kit (Qiagen), following the manufacturer’s instructions, and used for SNP and SSR genotyping. SNP genotyping was conducted commercially by Infinium Assay (Illumina, CA).

### Microscopic Observation

Stoma counting and aperture observations were performed using an imprinted slide. Briefly, both sides of flag leaf sheaths were coated with 10% cellulose acetate dissolved in acetone using a paint brush. When dried, the cellulose acetate film (∼2×1 cm) was carefully peeled, mounted on a slide, covered with a cover glass, and observed using a light microscope at a magnification of 10×20. Two imprints were taken from each side of the sheath and three independent inspections were carried out on each of the imprints.

For SEM imaging of cuticle surfaces, a 0.5-cm tissue fragment was harvested from the base of the flag leaf, the uppermost part of the flag leaf sheath, and the peduncle, and glumes were collected from the spikelets in the middle of spikes at anthesis (F10.5.1). The tissue samples went through 10 glycerol gradients, from 10% to 100%, with 2 h in each glycerol solution, to replace the cellular water. The pretreated samples were sputtered with gold powder using the CrC-150 Sputtering System, and inspected with a Hitachi S-3400N SEM (Hitachi). Images were captured with the voltage set at 5 kV.

### GC-MS Profiling of Wax Composition

Cuticular wax was extracted from two flag leaf sheaths of similar age from the same plant by submerging tissues in a glass tube containing 10 ml of HPLC grade chloroform (Fisher Scientific) and 4 µg of tetracosane (Sigma-Aldrich) as an internal standard and agitating manually for 1 min. The tissue was rinsed with an additional 5 ml of chloroform, and the two extracts were combined. The wax extract was filtered through a polytetrafluoroethylene filter with a 10-mL SGE syringe (Supelco) into a new glass tube and dried under a nitrogen stream. The dried wax was resuspended in 500 µl of acetonitrile and silylation was performed in 6% bis-trimethylsilyl-trifluoroacetamide and 10% trimethyl-chlorosilane at 80°C for 30 minutes to transform the hydroxyl and carboxylic groups into trimethylsilyl derivatives. The suspension was concentrated to 200 µl, and 1 µl was used for GC-MS analysis. Six biological replicates were included for each genotype. Wax silylation, GC-MS profiling, and substance identification were performed at the W.M. Keck Metabolomics Research Laboratory of Iowa State University (Ames, IA) on a fee-for-service basis.

### Quantification of Cuticle Traits

To evaluate the effect of cuticular wax on water loss rate, flag leaf sheaths were excised from wheat plants at stage F10.5.1, dehydrated for 12 h at room temperature with a relative humidity of 44%, and weighed every hour using an analytical balance with a readability of 0.001 mg. The dry weight of tissues was measured after incubation at 37°C for 72 h. For the chlorophyll efflux assay, a flag leaf sheath was placed in a 50-ml tube containing 30 ml of 80% ethanol and the tube was gently agitated on a rotator at 50 rpm. A 150-µl aliquot of chlorophyll extract was transferred to a well in a microplate for quantification and then returned to the same tube. Absorbance was measured at 647 and 664 nm using a Synergy 2 Multi-Mode Microplate Reader (Biotek) and total chlorophyll micromoles were calculated as described [75]. Two measurements were adopted as technical replicates and four biological replicates were included for both experiments.

### Transcription Quantification

Wheat cuticle gene homologs were identified by BLASTn searches of the wheat gene index database (http://compbio.dfci.harvard.edu/tgi) using known wax gene sequences as queries. The maximal cut-off value was set at E-20. qPCR primers were designed using the Primer3 program (http://frodo.wi.mit.edu/), with the PCR product size ranging from 80 to 200 bp (Table S1). Total RNA was isolated from flag leaf sheaths using Trizol® reagent (Invitrogen), following the manufacturer’s instructions. After RNA integrity evaluation by agarose gel electrophoresis and quantification using Nanodrop ND-1000 (Thermo Scientific), 1 µg of total RNA was used for cDNA synthesis in a 20-µl reaction using the QuantiTect Rev Transcription Kit (Qiagen). After dilution, ∼5 ng of cDNA was used as template for qPCR in a volume of 20 µl. qPCR was performed in 96-well plates with an ABI 7900HT High-Throughput Real-Time Thermocycler (Life Tech) using the iTaq™ Fast SYBR® Green Supermix with ROX (Bio-Rad). Two technical and four biological replicates were included for each NIL. The comparative ΔΔCT method was used to evaluate the relative quantities of each amplified product using *TaRPII36* as an internal reference in the same run. PCR specificity was determined by melt curve analysis of the amplified products.

### Data Analysis and Statistics

Measured values from replicates were averaged and their standard deviations (SD) were estimated using Microsoft® Excel functions. Student’s *t*-test was performed using pooled SDs to evaluate the statistical significance of the differences among isogenic lines. The cut-off for statistical significance was set to a *P*-value of 0.05 or less.

## Supporting Information

Figure S1 A diagram showing our current understanding of cuticular wax deposition.
(DOCX)

Figure S2 Homolog variation of major wax species among the NILs.
(DOCX)

Figure S3 Validation of reference genes.
(DOCX)

Figure S4 Transcriptional changes of fatty acyl elongation genes in *iw1Iw2* compared to *W1W2* at the seedling (F4.0) and adult plant (F9.0) stages.
(DOCX)

Table S1 qPCR primers designed from wheat ESTs homologous to the wax genes characterized in Arabidopsis, maize, and rice.
(DOCX)

Table S2 Transcription fold changes of cutin- and cuticular wax-related genes in *Iw1iw2* and *iw1Iw2* in comparison to *W1W2*.
(DOCX)

Table S3 Transcription fold changes of cutin- and cuticular wax-related genes in the glaucous NILs compared to *w1w2*.
(DOCX)
