# Supplementary material for: Genetic Interactions Underlying the Biosynthesis and Inhibition of β-Diketones in Wheat and Their Impact on Glaucousness and Cuticle Permeability
Source: PLoS One. 2013 Jan 17;8(1):e54129. doi: 10.1371/journal.pone.0054129 (PMC3547958; doi:10.1371/journal.pone.0054129)
Supplement: Figure S1 — A diagram showing our current understanding of cuticular wax deposition. (DOCX) [file pone.0054129.s001.docx]

**Figure S1.** A diagram showing our current understanding of cuticular wax deposition. Components of different wax pathways are distinguished by colors indicated at the upper left corner. The green pentagonal boxes indicate wax transporters. The short yellow lines indicate wax crystallites. While the acyl elongation, reduction and decarbonylation pathway is ubiquitous in the C3 and C4 plants, the β-diketone pathway is confined to some C3 plants, such as wheat and barley.
