# Supplementary material for: Genetic Interactions Underlying the Biosynthesis and Inhibition of β-Diketones in Wheat and Their Impact on Glaucousness and Cuticle Permeability
Source: PLoS One. 2013 Jan 17;8(1):e54129. doi: 10.1371/journal.pone.0054129 (PMC3547958; doi:10.1371/journal.pone.0054129)
Supplement: Figure S2 — Homolog variation of major wax species among the NILs. (DOCX) [file pone.0054129.s002.docx]

**Figure S2.** Homolog variation of major wax species among the NILs. Carbon atom numbers of fatty acids (a) and alkanes (b) are indicated on the x-axes. Their contents are indicated on y-axes as µg per g dried tissue (dry weight, DW). The bars indicate the deviation from the mean estimated from six biological replicates.
