# Supplementary material for: Genetic Interactions Underlying the Biosynthesis and Inhibition of β-Diketones in Wheat and Their Impact on Glaucousness and Cuticle Permeability
Source: PLoS One. 2013 Jan 17;8(1):e54129. doi: 10.1371/journal.pone.0054129 (PMC3547958; doi:10.1371/journal.pone.0054129)
Supplement: Figure S3 — Validation of reference genes. (DOCX) [file pone.0054129.s003.docx]

a

b

**Figure S3.** Validation of reference genes. Expression stability and ranking of reference genes as calculated by qBase^plus^ (a) and NormFinder (b) in all 18 cDNA samples. Both the M value (a) and stability value (b) in the y-axes are inversely proportional to the gene expression stability.
