# Supplementary material for: Genetic Interactions Underlying the Biosynthesis and Inhibition of β-Diketones in Wheat and Their Impact on Glaucousness and Cuticle Permeability
Source: PLoS One. 2013 Jan 17;8(1):e54129. doi: 10.1371/journal.pone.0054129 (PMC3547958; doi:10.1371/journal.pone.0054129)
Supplement: Figure S4 — Transcriptional changes of fatty acyl elongation genes in iw1Iw2 compared to W1W2 at the seedling (F4.0) and adult plant (F9.0) stages. (DOCX) [file pone.0054129.s004.docx]

**Figure S4.** Transcriptional changes of fatty acyl elongation genes in *iw1Iw2* compared to *W1W2* at the seedling (F4.0) and adult plant (F9.0) stages. The error bars indicate standard deviation of the average change estimated from four biological replicates.
