# Supplementary material for: Genetic Interactions Underlying the Biosynthesis and Inhibition of β-Diketones in Wheat and Their Impact on Glaucousness and Cuticle Permeability
Source: PLoS One. 2013 Jan 17;8(1):e54129. doi: 10.1371/journal.pone.0054129 (PMC3547958; doi:10.1371/journal.pone.0054129)
Supplement: Table S1 — qPCR primers designed from wheat ESTs homologous to the wax genes characterized in Arabidopsis, maize, and rice. (DOCX) [file pone.0054129.s005.docx]

| **Table S1**. qPCR primers designed from wheat ESTs homologous to the wax genes characterized in Arabidopsis, maize, and rice. | | | | | |
| --- | --- | --- | --- | --- | --- |
| Homologous genes | ESTs | Forward primer | Reverse primer | Size (bp) | Reference |
| Reference genes |  |  |  |  |  |
| *18s rRNA* | AY049040 | CGGAGAATTAGGGTTCGA | CCGTGTCAGGATTGGGTA | 97 | This paper |
| *ARF* | CK212331 | GCTCTCCAACAACATTGCCAAC | GCTTCTGCCTGTCACATACGC | 165 | Paolacci et al 2009 |
| *α tubulin* | DQ435669 | GCCATCTACGACATCTGCAA | GGTCTGGAACTCGGTTATGTCC | 150 | This paper |
| *H3* | CK211786 | GGAGATCCGCAAGTACCAGA | AGGTTGGTGTCCTCGAACAG | 180 | This paper |
| *Cdc48* | CJ685526 | GAGGAGGATGAGGTGGATGA | CCTGGTACTTGCGGATGTCT | 109 | This paper |
| *MetAP1* | CA680072 | AGTTGTGACCCGAGGAAAATC | GCGTCAGCAAATAGCAAGTG | 112 | This paper |
| *TaRPII36* | CJ665632 | ACGTATTAACCAAGAACTCATGGAGAC | TCAAATACTTTTGTAGGGCTGCTCTC | 84 | Xue et al. 2008 |
| *TaSAG8* | AB539589 | GGCCTTGAGATTGACGACAT | CTCCTCCATTCACGTTCACC | 118 | This paper |
| *TEF-1α* | CJ684593 | CGGTTGCTGTTGGTGTCATC | TCATTGCTCGCTCGGGATAA | 164 | This paper |
|  |  |  |  |  |  |
| Cutin biosynthesis |  |  |  |  |  |
| *ATT1/CYP86A2* | AF123609 | AGATAGGGCAGCAGTGCAAGA | TCTGGAAAGGAGTGGCTTCAA | 78 | Kosma et al. 2010 |
| *BDG* | CA653379 | CCACATGTACAACCCTACTACTGCTACT | CCATTGATCGCCTCCTCTCTT | 105 | Kosma et al. 2010 |
| *GPAT4* | CJ639155 | TCAAGCTCATGGACCCCTAC | TGTAGTTGGCGACCTCGAT | 129 | This paper |
| *HTH1* | CK152094 | GGACGAGCAGCTGGAGTGTAG | ACACTGGATCCCAGCAAAAATT | 100 | Kosma et al. 2010 |
| *LCR* | AF123609 | GTACAAGTTCGTGGCGTTCA | AGCGACATCTTCTGCTCCAC | 150 | This paper |
|  |  |  |  |  |  |
| Fatty acyl elongation |  |  |  |  |  |
| *ACC1* | U10187 | CACCAGGGCGTGAAAAGTAA | TAAGCGCCAGCCCAGAGTA | 139 | This paper |
| *FATB* | CJ854689 | CGGGGATAAATGGTTGATTG | ATTCCTCCAAAGCCTGTCAA | 161 | This paper |
| *CER10* | CA629021 | TCGCCGATGGGTGATTCTT | TTCGGACGCTGGAACAGAAC | 100 | Kosma et al. 2010 |
| *GL8* (maize) | CA624492 | CCGTCCGATCCACTCTACTC | CGAATAGCAGCACGAGCATA | 201 | This paper |
| *KCR1* | CJ850206 | TGCAATGCCAGGTGCCCTT | GGGAGATCAAGAACCACAGAAC | 171 | This paper |
| *KCR2* | CD453638 | CATCTCGGCTCGTAACCAAT | GATGTCCAATGCCAGATTCC | 131 | This paper |
| *KCS-1* | CJ803070 | CCCACATCCACAAGTAGCAG | GATCGGAGAGCAGAGCAGAG | 197 | This paper |
| *KCS-2* | CN010339 | CCACCTCTACCCGTCCAAGA | ATGCAGAAGTGCTCGAACG | 114 | This paper |
| *KCS-3* | BJ276980 | AGCTCTCTGGCTTTGCTTCC | GCCCGTTCGTTGAGATTTAC | 126 | This paper |
| *KCS-4* | CJ579236 | ATACCACCCGGAACAGCAAG | AGTGAACAGCACGACACCAC | 140 | This paper |
| *KCS-5* | CK210419 | TGTTCCAGGAGGAGGACAAG | CCGATGAGAGAGGTGAGGAA | 157 | This paper |
| *KCS1* | CK215050 | TCCTCAAGTCGCTGATGATG | CTCCATGTCCTTGTCCTCCA | 164 | This paper |
| *KCS2* | CK163968 | TACGCAAGCTCGTACCAAAG | CCATCGAGAAGAACCTGGAG | 109 | This paper |
| *KCS6* | AL820835 | ATGTTGAATTGGAGGACGCTAAC | CGCAGTGGAAATCCGTGACT | 75 | Kosma et al. 2010 |
| *LACS1* | CJ687277 | CCTTCTCGTGGTGAAATCCT | TCTCCTGTATGAAACCATCCAT | 110 | This paper |
| *LACS3* | CJ567199 | CAAGTACTACCAGGGCGCAAT | TTACACCACTCATCCGATCCA | 178 | This paper |
| *Wsl1*(rice) | CJ699046 | CACCCAGGAGGAGGATGATA | GATGCAGAAGTGCTCGAATG | 229 | This paper |
|  |  |  |  |  |  |
| Acyl reduction |  |  |  |  |  |
| *CER4-1* | AJ459251 | CCGATTCCGCATTCAACTTT | GACACCAGGGATGTGGACCTT | 77 | Kosma et al. 2010 |
| *CER4-2* | CD454643 | TTCCATTTGCATTGATCTGC | CCCCGGCTAGAATGCAAGTA | 199 | This paper |
| *CER4-3* | CJ671384 | GACCAAGCACGCAAGTACAG | TTCGGGTCGAAATTGAAGAG | 152 | This paper |
| *CER4-4* | CJ601183 | CAGGATGGGAATGAACAAGG | TACCGCACTGGGAATTTGTC | 172 | This paper |
| *CER4-5* | BE585519 | CAAGAGGGCACGACACTTTG | CTGATTCCTTCCATCCATCC | 168 | This paper |
| *CER4-6* | CV782109 | TGTGGGTTGTTTTCCCAATG | TGCCGTCCATCTTTACTTCC | 154 | This paper |
| *CER4-7* | BE446195 | GCTCTATGCGCCTTACACCT | GTCCCAGTCGATTTTCTTGG | 137 | This paper |
| *CER4-8* | CJ654933 | CCGTTGACTGGGACGATTAC | TTACACATGGTGGGCAACG | 159 | This paper |
| *CER4-9* | CV770866 | CCGCCACAACCAACTTCTAC | ACGTGCATGAGCACTTCGAG | 120 | This paper |
| *CER4-10* | AJ459251 | CTTTGACCCCAAGACCATTG | CCTCCATTCCTTCTCAGCAC | 127 | This paper |
| *CER4-11* | CK210480 | AGAGGGAGGCCATGAAGG | TCTGGACGCTGGTTATGATG | 140 | This paper |
| *CER4-12* | AJ459253 | TTAATTTCGACCCCAAGACC | TCGGTGCATGGATATTTCAG | 102 | This paper |
| *CER4-13* | CJ802718 | ACGTGTTCACCAAGGCAATG | GACTCCTTCCATCCATCCAG | 130 | This paper |
| *CER4-14* | CJ689207 | GGATGGGAATGAACGAGGAC | ATTTGAGCACACCAGGGATG | 120 | This paper |
| *FAR2* | CJ654714 | TAACACCGAACACCGAAATG | CTGCTAACACAGAACCAAACG | 108 | This paper |
| *FAR5* | CJ658504 | GACACTAAGGGAGGGCACAC | TGGTTCTCCTCTCAGCCTTC | 113 | This paper |
| *WSD1* | CK163540 | CAGAAGCCCTAACCGTGAAT | TGGATATGTTGCACTGATGC | 200 | This paper |
|  |  |  |  |  |  |
| Decarbonylation |  |  |  |  |  |
| *CER1-1* | CJ580697 | CGAATTGTTGATGGAAGTGG | ACGATTCATGATCACCTGGA | 156 | This paper |
| *CER1-2* | CJ683546 | CGTTCCAATATCGCAGTTCC | CTTCTCGGCAACCAGTTCTC | 126 | This paper |
| *CER1-3* | CA600374 | TGAGCGTGCCTAAAACACTG | ACTTTGTCCATGTCGAGCAC | 154 | This paper |
| *CER1-4* | CJ654775 | AGCTCGTCAGATGCCTTGTC | TCCCTCCCGCTCAATATCTC | 103 | This paper |
| *CER1-5* | CJ684510 | CGCCTCCATAGCATCCAAAC | ATTCTCTCGACCACGAATGC | 126 | This paper |
| *CER1-6* | CV769999 | CGCATAGCCTCAATGGAAGTG | CGGGGAATAGTGTGGATGAC | 116 | This paper |
| *CER1-7* | CJ687274 | GCAGAACATCCACTCCTGTG | CTTTGGTGAGAGGGACGAAG | 179 | This paper |
| *CER1-8* | CJ686958 | GATCATGTGGCCTCTGTCCT | CTTGGTATGGCCCAAGACTG | 117 | This paper |
| *MAH1-1* | BQ243104 | AGTCTCCATTCCCCAACTCC | AGAAGACGACGAAGCAGAGC | 141 | This paper |
| *MAH1-2* | CJ577491 | GAGCTGGTGGAAGGACAGAG | TATGGACACACAGACATACGC | 156 | This paper |
| *MAH1-3* | BQ237796 | AAGGAACGGAATGTGGTACAG | GCACAAGGAGCACACATACG | 188 | This paper |
| *MAH1-4* | BE430508 | GCGCGTAGAAGATCAGTAGACC | CTGTGGTGTGTTGGACTCGT | 139 | This paper |
| *MAH1-5* | CJ661998 | AACGACGAGGAACAAGATGG | TAGGGTTCTGGGTGAGGTTG | 169 | This paper |
| *MAH1-6* | CA597553 | TCCACGACTGGGTCACCACT | AGTTGGCGAAGTTGGAGGTG | 141 | This paper |
| *MAH1-7* | CN010705 | CATCCAAACTTCTCCACTACACC | TCCTTGCATGTTTTCTTGGAC | 109 | This paper |
| *MAH1-8* | CJ672121 | TGACGTGCAACCTCGTCTTC | ACGTGCCGGAGGAAGGTG | 115 | This paper |
| *CER3-1* | CA594529 | TGACGCCGGTGTGATGTG | CGATCTTAGTCGCTTCGTTTCA | 82 | Kosma et al. 2010 |
| *CER3-2* | CJ683887 | AGAACCACCTCATCCTGCTG | CGGAGGTAGTAGAAGCTGACG | 103 | This paper |
| *CER3-3* | CK171682 | CATCAACCGCCAGATCGAG | CTCAGGTCAGGGTGCTTGTC | 138 | This paper |
| *CER3-4* | CJ685307 | AGATCGCTCCCTCCTCCATC | GTGTTGTCCTTGGGTGGTTC | 116 | This paper |
| *CER3-5* | BJ209879 | CGTGCGTGTGTTGATACTTG | TCGAAACGATCTCCCATCAC | 164 | This paper |
|  |  |  |  |  |  |
| Transporters |  |  |  |  |  |
| *WBC11* | CN011458 | ATGTCGGCACCAAATACACA | CGCTGGAAAACCTTCATTTC | 127 | This paper |
| *WBC15* | CK209928 | GGGGTTTGAAGGGTGCATA | TAGGCCAGGAGGAGGATGA | 172 | This paper |
| *WBC19* | CJ600721 | CTCTTCAGCGGCTTCTTCAT | GGCGTGTTGTCGAACATCT | 164 | This paper |
| *ABCG15* | CJ676748 | GGGGTTTGAAGGGTGCATA | GCCAGGAGGAGGATGAAGAT | 169 | This paper |
| *LTP* | AL828316 | GGTGCCTCTGCTCCTACAAG | GAGGGTGGATGGATCTAGCA | 127 | This paper |
| *LTP1* | CA630456 | AATGGTCCTCACAGCCACA | CCAGACTCCTGACACCACTG | 137 | This paper |
| *LTP4* | AF302788 | CCGGAAAGGCTGCAAGTATC | ACGGATGCGCTGATGGA | 67 | Kosma et al. 2010 |
|  |  |  |  |  |  |
| Regulators |  |  |  |  |  |
| *CER7* | BJ275196 | GTGCAATGGAAAACCAAACC | CTGACTCTCCAGCACTTCCA | 168 | This paper |
| *MYB30* | CJ664139 | GTGAAGAGGCTGCAACAACA | GGCAGAGTCGAGGTTACTGC | 132 | This paper |
| *MYB96* | JF951907 | GTCGCTGTCCCAGTGCTC | CGCTGCCGTAGGAAACCTC | 161 | This paper |
| *Ocl1* (maize) | CJ826679 | CCATGAGTGCCAACCAGAG | AAATCCAGATGGCAGCAGAG | 158 | This paper |
| *WIN1/SHN* | CK164430 | AGAAGTTCCGTGGAGTCAGG | CACGGGGAAGTTGGTCTTG | 179 | This paper |

**Kosma DK, Nemacheck JA, Jenks MA, Williams CE** (2010) Changes in properties of wheat leaf cuticle during interactions with Hessian fly. Plant J. 63:31-43.
