# Supplementary material for: Genetic Interactions Underlying the Biosynthesis and Inhibition of β-Diketones in Wheat and Their Impact on Glaucousness and Cuticle Permeability
Source: PLoS One. 2013 Jan 17;8(1):e54129. doi: 10.1371/journal.pone.0054129 (PMC3547958; doi:10.1371/journal.pone.0054129)
Supplement: Table S2 — Transcription fold changes of cutin- and cuticular wax-related genes in Iw1iw2 and iw1Iw2 in comparison to W1W2. (DOCX) [file pone.0054129.s006.docx]

| **Table S2.** Transcription fold changes of cutin- and cuticular wax-related genes in *Iw1iw2* and *iw1Iw2* in comparison to *W1W2*^1^ | | |
| --- | --- | --- |
| Cuticle genes | *Iw1iw2* | *Iw1Iw2* |
| Cutin biosynthesis | | |
| *ATT1/CYP86A2* | 1 | -1.25 |
| *BDG* | -1.09 | 1.14 |
| *GPAT4* | -1.02 | -1.01 |
| *HTH1* | 1.08 | 1.32^*^ |
| *LCR/CYP86A8* | 1.08 | -1.24 |
|  |  |  |
| Fatty acid elongation | | |
| *ACC1* | -1.11 | 1.39 |
| *CER10/ECR* | 1.06 | 1.77^**^ |
| *FATB* | -1.03 | -1.03 |
| *GL8* | 1.3 | 1.95^**^ |
| *KCR1* | -1.21 | 1.19 |
| *KCR2* | -1.1 | 1.16 |
| *KCS-1* | 1.05 | 1.11 |
| *KCS-2* | -1.13 | 1.32 |
| *KCS-3* | -1.02 | -1.39^*^ |
| *KCS-4* | -1.17 | -1.44^**^ |
| *KCS-5* | 1.08 | 1.86^**^ |
| *KCS1* | 1.12 | 1.86^**^ |
| *KCS2/DAISY* | 1.03 | 1.50^**^ |
| *KCS6/CER6* | 1.13 | 1.37 |
| *LACS1/CER8* | -1.15 | 1.33 |
| *LACS3* | 1.39 | 1.70^**^ |
| *WSL1* | 1.06 | -1.08 |
|  |  |  |
| Acyl-CoA reduction | | |
| *CER4-1* | -2.04^**^ | -1.69^**^ |
| *CER4-2* | -1.05 | -1.34 |
| *CER4-3* | 1.36 | 2.19^**^ |
| *CER4-4* | -1.19 | 1.63^**^ |
| *CER4-5* | -1.46 | 1.90^*^ |
| *CER4-6* | 2.79^*^ | 119.45^**^ |
| *CER4-7* | -1.07 | 1.18 |
| *CER4-8* | -1.33^*^ | -1.28 |
| *CER4-9* | -1.01 | -1.07 |
| *CER4-10* | -1.26 | -1.21 |
| *CER4-11* | 2.26^**^ | 2.08^**^ |
| *CER4-12* | 1.03 | 1.62^*^ |
| *CER4-13* | -1.15 | 1.36^*^ |
| *CER4-14* | -1.26 | 1.3 |
| *FAR2* | -1.01 | -1.01 |
| *FAR5* | -1.05 | -1.07 |
| *WSD1* | -1.31 | 1.74^**^ |
|  |  |  |
| Decarbonylation | | |
| *CER1-1* | -1.12 | -1.11 |
| *CER1-2* | -1.15 | -1.21 |
| *CER1-3* | -1.33 | 1.34 |
| *CER1-4* | -1.11 | -1.35 |
| *CER1-5* | -1.14 | -1.17 |
| *CER1-6* | -1.62^*^ | 1.45^*^ |
| *CER1-7* | 1.09 | -1.14 |
| *CER1-8* | 1.77^*^ | 2.12 |
| *MAH1-1* | 1.21 | 1.85 |
| *MAH1-2* | -1.47 | -1.81^*^ |
| *MAH1-3* | 2.82^*^ | 1.03 |
| *MAH1-4* | -1.08 | -1.07 |
| *MAH1-5* | -1.76^**^ | -1.01 |
| *MAH1-6* | 1.43 | 2.58^**^ |
| *MAH1-7* | 1.11 | 3.87^*^ |
| *MAH1-8* | 1.58^*^ | 9.96^**^ |
| *CER3-1* | 1.01 | 1.1 |
| *CER3-2* | 1.12 | 1.43^**^ |
| *CER3-3* | 1.1 | 1.48^*^ |
| *CER3-4* | 1.04 | 1.07 |
| *CER3-5* | 1.13 | 1.3 |
|  |  |  |
| Transporters | | |
| *ABCG11* | 1 | 1.56^**^ |
| *ABCG15* | -1.03 | 1.78^**^ |
| *ABCG19* | 1.05 | 1.44 |
| *ABCG31* | 1.02 | 1.13 |
| *LTP* | 1.06 | 1.38 |
| *LTP1* | 1.03 | -1.04 |
| *LTP4* | 1.15 | 2.04^**^ |
|  |  |  |
| Regulators | | |
| *CER7* | -1 | -1.34^*^ |
| *MYB30* | -1.12 | 1.21 |
| *MYB96* | -1.07 | 1.13 |
| *OCL1* | 1.07 | 1.11 |
| *WIN1/SHN1* | 1.49^*^ | 1.25^*^ |

^1^ Asterisks indicate that the difference is significant at *P*< 0.05 (*) or at *P* < 0.01 (**).
