# Supplementary material for: Genetic Interactions Underlying the Biosynthesis and Inhibition of β-Diketones in Wheat and Their Impact on Glaucousness and Cuticle Permeability
Source: PLoS One. 2013 Jan 17;8(1):e54129. doi: 10.1371/journal.pone.0054129 (PMC3547958; doi:10.1371/journal.pone.0054129)
Supplement: Table S3 — Transcription fold changes of cutin- and cuticular wax-related genes in the glaucous NILs compared to w1w2. (DOCX) [file pone.0054129.s007.docx]

| **Table S3.** Transcription fold changes of cutin- and cuticular wax-related genes in the glaucous NILs compared to *w1w2*^1^ | | | |
| --- | --- | --- | --- |
| Wax genes | *W1w2* | *w1W2* | *W1W2* |
| Cutin biosynthesis | | | |
| *ATT1/CYP86A2* | -1.26 | -1.18 | -1.09 |
| *BDG* | 1.03 | 1.05 | -1.03 |
| *GPAT4* | 1.02 | -1.09 | 1.02 |
| *HTH1* | 1.04 | 1.05 | -1.16 |
| *LCR/CYP86A8* | -1.01 | -1.2 | 1.08 |
|  |  |  |  |
| Fatty acyl elongation | | | |
| *ACC1* | 1.03 | 1.06 | -1.02 |
| *CER10/ECR* | -1.03 | -1.07 | -1.22^*^ |
| *FATB* | -1.09 | -1.15 | -1.12 |
| *GL8* | -1.15 | -1.22^**^ | -1.46^**^ |
| *KCR1* | -1.02 | -1.09 | -1.17^*^ |
| *KCR2* | 1.07 | 1.01 | -1.14 |
| *KCS-1* | -1.14 | -1.01 | -1.15 |
| *KCS-2* | 1.2 | -1 | 1.06 |
| *KCS-3* | -1.03 | -1 | 1.15 |
| *KCS-4* | -1.3 | -1.24 | -1.17 |
| *KCS-5* | 1.12 | -1.48 | -1.42^**^ |
| *KCS1* | 1.13 | -1.07 | -1.17 |
| *KCS2/DAISY* | -1.09 | -1.39^*^ | -1.73^**^ |
| *KCS6/CER6* | -1.35 | -1.32^*^ | -1.66^**^ |
| *LACS1/CER8* | 1.08 | 1.04 | 1.09 |
| *LACS3* | -1.11 | 1.06 | -1.33^**^ |
| *WSL1* | -1.29 | -1.28 | -1.08 |
|  |  |  |  |
| Acyl-CoA reduction | | | |
| *CER4-1* | -1.33 | 1.03 | -1.12 |
| *CER4-2* | 1.14 | -1.58 | 1.29 |
| *CER4-3* | -1.88 | -1.89^**^ | -2.87^**^ |
| *CER4-4* | 1.92^*^ | -1.13 | -2.56^*^ |
| *CER4-5* | -1.84 | -2.26^*^ | -6.18^**^ |
| *CER4-6* | -1.07 | -1.72 | -3.83^*^ |
| *CER4-7* | 1.22 | -1.39^*^ | -1.39^**^ |
| *CER4-8* | -1.26 | -1.19 | 1.24 |
| *CER4-9* | -1.2 | -1.1 | 1.3 |
| *CER4-10* | 1.13 | 1.33 | 1.22 |
| *CER4-11* | 1.47 | 1.58 | 1.56 |
| *CER4-12* | -1.24 | -1.93^*^ | -1.83^*^ |
| *CER4-13* | -1.18 | -1.49^*^ | -1.26 |
| *CER4-14* | 1.70^**^ | -1.45^*^ | -1.67^**^ |
| *FAR2* | 1.41 | -1.1 | -1.3 |
| *FAR5* | -1 | 1.17 | 1.02 |
| *WSD1* | -1.34^*^ | -1.43^*^ | -1.98^**^ |
|  |  |  |  |
| Decarbonylation | | | |
| *CER1-1* | 1.22 | 1.38^*^ | 1.29^*^ |
| *CER1-2* | 1.12 | 1.26 | 1.02 |
| *CER1-3* | -1.67 | -1.99 | -1.49 |
| *CER1-4* | 1.15 | 1.04 | 1.08 |
| *CER1-5* | 1.37 | 1.15 | 1.26 |
| *CER1-6* | -1.39 | -1.68 | -1.04 |
| *CER1-7* | 1.27 | 1.26 | 1.17 |
| *CER1-8* | -2.05 | 2.60^*^ | -1.31 |
| *MAH1-1* | -1.21 | -1.09 | -2.23^*^ |
| *MAH1-2* | -1.05 | 1.17 | -1.06 |
| *MAH1-3* | -1.4 | 1.56 | 1 |
| *MAH1-4* | 1.06 | -1.16 | 1.06 |
| *MAH1-5* | 1.05 | -2.99^*^ | -1.36^**^ |
| *MAH1-6* | 1.57 | -1.07 | -1.27 |
| *MAH1-7* | -1.36 | -3.30^*^ | -1.1 |
| *MAH1-8* | 1.17 | 1.43 | 6.91^**^ |
| *CER3-1* | -1.05 | -1.15 | -1.51^**^ |
| *CER3-2* | 1.02 | -1.38^*^ | -1.34^*^ |
| *CER3-3* | 1.04 | -1.51^*^ | -1.38^*^ |
| *CER3-4* | -1.03 | -1.2 | -1.37 |
| *CER3-5* | 1.15 | 1.28 | -1.01 |
|  |  |  |  |
| Transporters | | | |
| *ABCG11* | -1.05 | -1.13 | -1.29 |
| *ABCG15* | -1.16 | 1.07 | -1.23 |
| *ABCG19* | -1.16 | -1.17 | -1.1 |
| *ABCG31* | 1.22 | 1 | 1.1 |
| *LTP* | 1.53^*^ | 1.51^*^ | 1.06 |
| *LTP1* | -1.32 | -1.85^*^ | -1.69 |
| *LTP4* | -1.36 | -1.54^*^ | -1.80^*^ |
|  |  |  |  |
| Regulators | | | |
| *CER7* | 1.02 | 1.06 | 1.09 |
| *MYB30* | -1.03 | -1.22 | -1.28 |
| *MYB96* | 1.06 | -1.55^*^ | -1.57^*^ |
| *OCL1* | 1.11 | 1.04 | 1.03 |
| *WIN1/SHN1* | -1 | 1.16 | -1 |

^1^ Asterisks indicate that the difference is significant at *P*< 0.05 (*) or at *P* < 0.01 (**).
